# Navigating the Systemic Conditions of a Digital Health Ecosystem in Alberta, Canada: Embedded Case Study

**DOI:** 10.2196/36265

**Published:** 2022-12-21

**Authors:** Chad Saunders, Devon Currie, Shane Virani, Jill De Grood

**Affiliations:** 1 Entrepreneurship & Innovation Haskayne School of Business University of Calgary Calgary, AB Canada; 2 Ward of the 21st Century Cumming School of Medicine University of Calgary Calgary, AB Canada

**Keywords:** digital health, entrepreneurial ecosystem, systemic conditions, policy

## Abstract

**Background:**

Digital health promises numerous value-creating outcomes. These include improved health, reduced costs, and the creation of lucrative markets, which, in turn, provide high-quality employment, productivity growth, and a climate that attracts investment. For this value creation and capture, the activities of a diverse set of stakeholders within a digital health ecosystem require coordination. However, the antecedents of the coordination needed for an effective digital health ecosystem are not well understood.

**Objective:**

The purpose of this study was to investigate the systemic conditions of the digital health ecosystem in Alberta, Canada, as critical antecedents to ecosystem coordination from the perspective of the authors as applicants to an innovative digital health funding program embedded within the larger digital health ecosystem of innovators or entrepreneurs, health system leaders, support partners, and funders.

**Methods:**

We employed a qualitative embedded case study of the systemic conditions within the digital health ecosystem in Alberta, Canada (main case) using semistructured interviews with 36 stakeholders representing innovators or entrepreneurs, health system leaders, support partners, and funders (subcases). The interviews were conducted over a 2-month period between May 26 and July 22, 2021. Data were coded for key themes and synthesized around 5 propositions developed from academic publications and policy reports.

**Results:**

The findings indicated varying levels of support for each proposition, with moderate support for accessing real problems, data, training, and space for evaluations. However, the most fundamental gap appears to be in ecosystem navigation, in particular, the absence of intermediaries (eg, individuals, organizations, and technology) to provide guidance on the available support services and dependencies among the various ecosystem actors and programs.

**Conclusions:**

Navigating the systemic conditions of the digital health ecosystem is extremely challenging for entrepreneurs, especially those without prior health care experience, and this remains an issue even for those with such experience. Policy interventions aimed at increasing collaboration among ecosystem support providers, along with tools and incentives to ensure coordination, are essential as the ecosystem and those dependent on it grow.

## Introduction

### Background

Alberta Innovates is Alberta’s largest research and innovation agency. In the fall of 2020, they launched a new initiative called the Health Innovation Platform Partnerships (HIPP). The objective of HIPP was “...to build a health innovation ecosystem that is robust, coordinated, and a competitive advantage for Alberta innovators in the health industry” [1]. Recognizing the need to foster more coordination among ecosystem stakeholders, the approach taken with this initiative was quite different from prior funding programs. In this regard, the grant was structured with incentives for applicants to not only provide value-generating activities to clients but also incentivize coordination with other ecosystem actors. There were 2 stages of funding, with the initial stage being an open call for proof-of-concept proposals. These were narrowed down to 11 applicants, who were then invited to submit a full application 6 months later at stage 2, informed by their stage 1 findings. What follows is a case analysis of the experience of one of the applicants, as they validated key ecosystem assumptions underlying their proposed initiative. Specifically, the authors constituted a team at the University of Calgary that submitted an application for a Digital Health Collaboratorium as part of the Ward of the 21st Century (W21C) Research and Innovation Centre, an established health systems research initiative with an overarching mandate to improve patient safety and quality of care [2].

### Entrepreneurial Ecosystems

**Figure 1:**
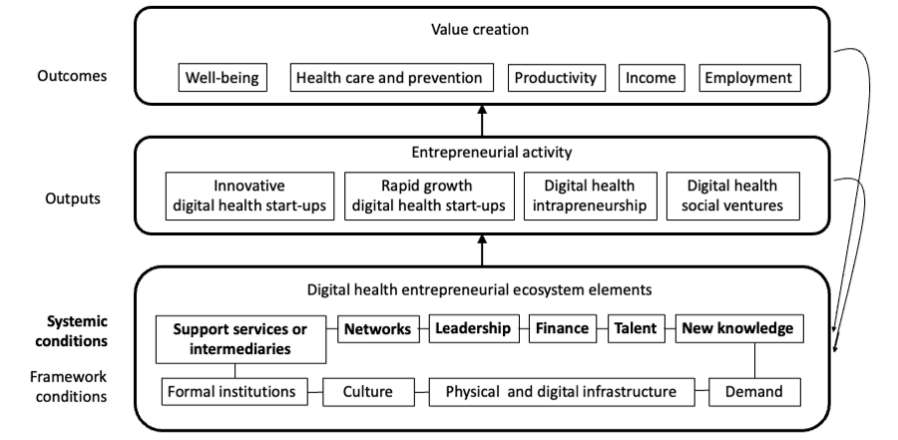
Theoretical model of digital health entrepreneurial ecosystems.

Entrepreneur ecosystems are defined as “a set of interdependent actors and factors coordinated in such a way that they enable productive entrepreneurship” [3]. The assessment of digital health entrepreneurial ecosystems is based on a reconceptualization of the theoretical model developed by Stam and Spigel [4], as shown in Figure 1. Building on a comprehensive review of existing research, including related concepts such as industrial districts, clusters, and innovation systems, Stam [3] created a theoretical model containing 10 key ecosystem elements. The framework conditions included social (ie, informal and formal institutions) and physical conditions enabling or constraining entrepreneurial activity. Systemic conditions are at the core of the ecosystem, representing networks of entrepreneurs, leadership, finance, talent, knowledge, and support services. The presence of these elements and the interactions between them predominantly determine the success of the ecosystem [3] and thus serve as the focus of this study.

As Stam [3] points out, networks of entrepreneurs facilitate information flow, which, in turn, enables the effective distribution of people and funding. Leadership provides direction and role models for the entrepreneurial ecosystem and is critical for establishing and maintaining ecosystem health. Access to finance is crucial to support the ongoing entrepreneurial activities with their inherent risks and fuel a diverse and skilled group of talented workers. Finally, a supply of support services by a variety of intermediaries can substantially lower the entry barriers for new entrepreneurial projects and facilitate product and service introduction.

Stam and Spigel [4] explained the following:

The new model includes insights from the previous literature (i.e., the aspects that have been deemed important elements of entrepreneurial ecosystems), but most importantly it provides more causal depth...including the upward and downward causation, and intra-layer causal relations. Upward causation reveals how the fundamental causes of new value creation are mediated by intermediate causes, while downward causation shows how outcomes and outputs of the system over time also feed back into the system conditions.

We foreground the systemic conditions as critical antecedents of entrepreneurial activity within the digital health entrepreneurial ecosystem. The purpose of this study was to explore a set of propositions based on the systemic conditions within a digital health ecosystem for key stakeholders, including digital health innovators or entrepreneurs, investors, and health system leaders.

Digital health promises value creation outcomes, including improved health, reduced costs, and the creation of lucrative markets, which, in turn, provide high-quality employment, productivity growth, and attract investment [5]. However, our understanding of how this occurs within entrepreneurial ecosystems generally [3] and within a digital health ecosystem specifically remains limited [6]. The theoretical model (Figure 1) indicates that for value creation and capture, the activities of a diverse set of stakeholders within a digital health ecosystem would need coordination. However, the antecedents of the coordination needed for an effective digital health ecosystem are not well understood [6].

### Proposition Development

#### Overview

This work was conducted through the O’Brien Institute for Public Health’s W21C Research and Innovation Centre at the University of Calgary. W21C serves as a research and beta test site for novel approaches to health care delivery, human factors research, and innovative medical technologies. Experience with health care innovators and entrepreneurs through prior work at W21C provided insight into the struggle of identifying real problems within the health care system. Even if problems are deemed legitimate, there is a further obstacle of prioritizing them against one another. It is very challenging for innovators and entrepreneurs to gain visibility into where problem-solving opportunities exist, and this is exacerbated if the innovator or entrepreneur is from outside the health domain. This issue has been broadly recognized within Alberta; in 2012, the Strategic Clinical Networks (SCNs) were created to bring together a diverse set of stakeholders to both identify and rank problems to facilitate the implementation of solutions [7]. Similar challenges have been identified in other jurisdictions of Canada. In Ontario, the approach of “build it and they will come” used by many innovators and entrepreneurs is not working for any of the ecosystem stakeholders [6]. Ultimately, it leads to solutions for nonproblems or solutions that are not feasible to implement in practice. This is an area where digital health is seen as a facilitator of the coordination needed and where the emergence of digital platforms in health care that effectively mediate 2-sided markets by providing technology that connects those that need a service with those that deliver the service lags behind other industries where these digital platforms are well established [8]. This leads to our first proposition.

#### Proposition 1: Digital Health Innovators and Entrepreneurs Struggle to Access Real Problems

Digital health innovators and entrepreneurs are often tasked with developing and implementing new digital health interventions to achieve better health outcomes, among other benefits. However, a critical challenge within health care—specifically for digital health technologies—is how to demonstrate *better* and the implications of these improvements for those who adopt the new technology. Evaluation approaches such as randomized control trials, which are required in other health technology assessments, are often impractical or not applicable to digital health innovations [9]. Although institutions such as the World Health Organization provide useful guidelines for monitoring and evaluating digital health interventions [10], most interventions require access to health care data that the innovator or entrepreneur lacks. This is often exacerbated by disconnects between health information legislation and consumer expectations around privacy and security surrounding the use of their data for digital health interventions, as these are increasingly more likely to be operated by companies such as Apple or Oracle rather than traditional health care providers [11,12]. The data generated using digital health technologies are mediated by a plethora of businesses, such as software or device companies, apps, and cloud hosting services, whose information governance processes are complicated and can further obscure visibility into the data needed to guide innovation [12]. This leads to our second proposition.

#### Proposition 2: Digital Health Innovators and Entrepreneurs Struggle to Access Relevant Data

As noted earlier, although organizations such as the World Health Organization provide guidance on the evaluation of digital health technologies [10] and various regulatory bodies have streamlined their review of specific digital health technologies (eg, Federal Drug Administration [13] and Health Canada [14]), getting help often remains elusive for many innovators and entrepreneurs. Ironically, the proliferation of Academic Medical Centers (AMCs), incubators, accelerators, and regional economic development initiatives focused on helping innovators and entrepreneurs has led to the unintended consequence of making it *more* difficult to navigate these increasingly complex and specialized ecosystem offerings [15].

The adoption of digital technologies in health care is additionally slowed by complex bureaucracy, laborious administrative approval processes, excessive risk assessment, understaffed health information technology departments, and a general reluctance to implement new apps in the clinical workflow. This makes the health care ecosystem incredibly complex and difficult to navigate [16]. Furthermore, entrepreneurs and innovators must identify and collaborate with clinical end users at the site (eg, physicians, nurses, etc) while also designing their solutions to concurrently satisfy pain points for a variety of other decision makers (eg, procurement specialists, practice managers, patients, health IT departments, and security or privacy officers) [16]. Thus, innovators and entrepreneurs are often at a loss to understand how to navigate the business side of their enterprise while also facing the dual challenge of navigating the health care system. This leads to our third proposition.

#### Proposition 3: Digital Health Innovators and Entrepreneurs Struggle to Understand Where to Start and Where They Need to Go on Their Innovation Journey

A critical systemic condition of an entrepreneurial ecosystem is the provision of talent, which in the context of digital health necessitates personnel trained in digital health to realize the expected improvement in outcomes [17]. There is a further need to increase interorganizational knowledge sharing to facilitate the creation of digital health learning ecosystems. This highlights that the knowledge and experience surrounding technology adoption and implementation is particularly valuable for members of other organizations contemplating similar digitally enabled transformations [18]. Although digital health learning ecosystems rely on formal mechanisms and processes to provide their foundation, they are most effective when supported by informal networks [18]. This leads to our fourth proposition.

#### Proposition 4: The Breadth of Training Provided Does Not Adequately Meet the Needs of Digital Health Innovators and Entrepreneurs

Emerging digital technologies offer enormous potential to improve quality, reduce costs, and increase patient centeredness in health care. Although AMCs play a key role in advancing medical care through cutting-edge medical research, traditional models for invention, validation, and commercialization have been designed around biomedical initiatives at AMCs. This makes them unsuitable for new digital health technologies [19]. However, AMCs are uniquely positioned. They house cross-disciplinary expertise in health and technology and train the next generation of health professionals, thus providing the opportunity to connect academics and clinicians from across a variety of disciplines with innovators and entrepreneurs [15]. This leads to our fifth proposition.

#### Proposition 5: Digital Health Innovators and Entrepreneurs Lack a “Space” to Evaluate Their Offerings

In the next section, we have presented an embedded case study to evaluate the set of propositions developed earlier based on the systemic conditions within a digital health ecosystem for key stakeholders, including digital health innovators or entrepreneurs, investors, and health system leaders.

## Methods

### Recruitment

To explore these propositions, a qualitative embedded case study of the systemic conditions within the digital health ecosystem in Alberta was conducted using semistructured interviews with 36 stakeholders, representing 31% (11/36) innovators or entrepreneurs, 19% (7/36) health system leaders, 39% (14/36) support partners, and 11% (4/36) funders (Table 1). These roles were selected using a theoretical sampling approach to match the systemic conditions of the entrepreneurial ecosystem framework guiding this study (Figure 1).

Participants were recruited from within the University of Calgary network, partnered organizations, and the community at large, leveraging the diverse connections of the W21C. The participants were selected using theoretical sampling based on their roles, interests, and goals in the development of digital health solutions, as guided by the theoretical framework (Figure 1). To ensure adequate coverage, the participants were also selected to cover a range of organizational sizes, activity scopes, and venture phases of entrepreneurs or support providers (Table 1). Interviews were conducted over a 2-month period between May 26 and July 22, 2021, by trained researchers following a pilot-tested semistructured interview protocol. The interviews were recorded (with consent from the participants) and then transcribed verbatim for data analysis. Thematic analysis methods were used on the interview data to address each proposition, and the resulting themes were synthesized using a Gioia data structure diagram [20].

Throughout the analysis, we were attuned to the presence or absence of particular aspects of the underlying assumptions that the participants raised and the depth of insight that they provided as indicators of whether a particular theme was relevant. Our qualitative approach focused on theoretical generalizability and not statistical generalizability. As such, themes were relevant based on a number of criteria, including their prevalence (ie, counts), the depth of coverage (including the time spent on the topic as a proxy for the importance) that the participants afforded the topic, and our assessment of the theoretical relevance of the topic in providing novel or nuanced understanding of the underlying assumption we were attempting to evaluate.

The findings from this study have been presented from the perspective of the authors as applicants to the innovative digital health funding program (ie, HIPP) embedded within the larger digital health ecosystem of innovators or entrepreneurs, health system leaders, support partners, and funders.

**Table 1:** Summary of the study participants by ecosystem role.

ID	Ecosystem role	Size	Scope	Phase
P02	Innovator or entrepreneur	Medium	International	Established
P03	Innovator or entrepreneur	Micro	AB^a^	Startup
P04	Innovator or entrepreneur	Micro	AB	Startup
P08	Innovator or entrepreneur	Small	National	Startup
P09	Innovator or entrepreneur	Small	National	Established
P11	Innovator or entrepreneur	Medium	International	Established
P14	Innovator or entrepreneur	Micro	AB	Startup
P15	Innovator or entrepreneur	Micro	AB	Startup
P18	Innovator or entrepreneur	Small	National	Startup
P21	Innovator or entrepreneur	Small	International	Startup
P31	Innovator or entrepreneur	Small	International	Startup
P24	Health system leader	Large	AB	Hospital or community
P25	Health system leader	Large	AB	Hospital
P26	Health system leader	Large	AB	Hospital
P28	Health system leader	Large	NS^b^	Hospital or community
P33	Health system leader	Large	BC^c^	Hospital or community
P35	Health system leader	Large	ON^d^	Hospital or community
P36	Health system leader	Large	NL^e^	Hospital or community
P01	Support partners	Small	National	All
P05	Support partners	Small	Alberta	All
P06	Support partners	Large	International	All
P07	Support partners	Small	International	Startup
P10	Support partners	Small	AB	Startup
P12	Support partners	Micro	AB	Startup
P13	Support partners	Large	AB	Established
P19	Support partners	Small	AB	Startup or rapid growth
P22	Support partners	Small	AB	All
P23	Support partners	Small	AB	All
P29	Support partners	Small	AB	Startup or rapid growth
P30	Support partners	Small	AB	Startup or rapid growth
P32	Support partners	Small	AB	Startup or rapid growth
P34	Support partners	Small	National	Startup or rapid growth
P16	Funders	Small	National	Startup or rapid growth
P17	Funders	Small	AB	Startup or rapid growth
P20	Funders	Small	AB	Rapid growth
P27	Funders	Large	National	Startup or rapid growth

^a^AB: Alberta.

^b^NS: Nova Scotia.

^c^BC: British Columbia.

^d^ON: Ontario.

^e^NL: Newfoundland and Labrador.

### Ethical Considerations

This study involved interaction with human participants and was approved by the University of Calgary’s Conjoint Faculties Research Ethics Board under the ethics ID REB21-0242. All the extracted data were anonymized before being analyzed. The participants were not compensated, and their participation was entirely voluntary.

## Results

### Overview

**Figure 2:**
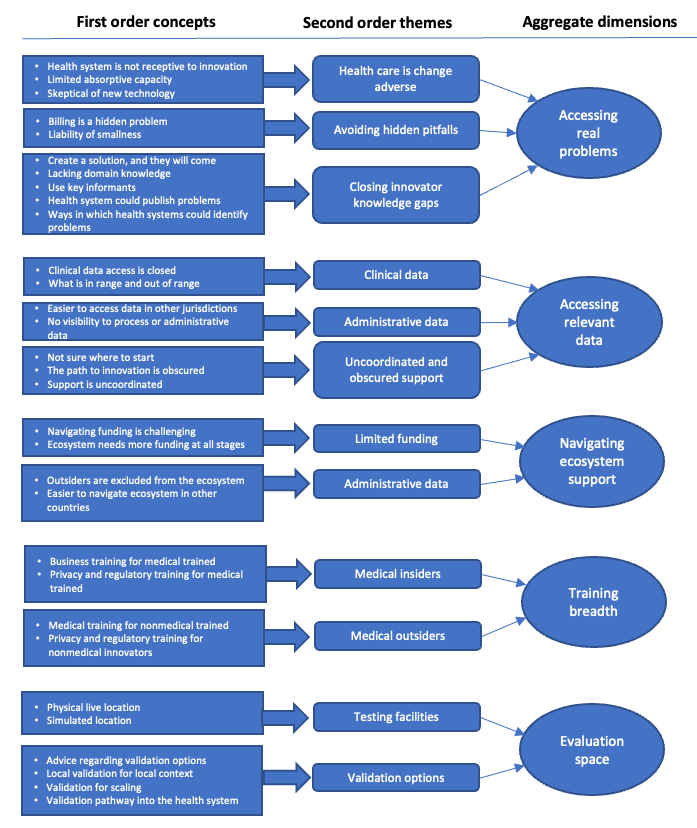
Data structure.

The findings have been organized around 5 propositions developed from the broader literature on the navigation of ecosystems by entrepreneurs, within the context of digital health. These propositions have been presented as aggregate dimensions supported by first-order concepts and second-order themes (Figure 2) and summarized using a Gioia data structure diagram [20].

Table 2 provides a summary of the level of support for the findings described in detail in the subsequent section.

**Table 2:** Summary of the level of support for the propositions.

Proposition	Level of support
Proposition 1: digital health innovators and entrepreneurs struggle to access real problems	Moderate
Proposition 2: digital health innovators and entrepreneurs struggle to access relevant data	Moderate
Proposition 3.: digital health innovators and entrepreneurs struggle to understand where to start and where they need to go on their innovation journey	Strong
Proposition 4: the breadth of training provided does not adequately meet the needs of digital health innovators and entrepreneurs	Moderate
Proposition 5: digital health innovators and entrepreneurs lack a “space” to evaluate their offerings	Moderate

### Proposition 1: Digital Health Innovators and Entrepreneurs Struggle to Access Real Problems

We anticipated that there would be a discrepancy between what digital health innovators or entrepreneurs and health system leaders perceive as problems. This disconnect would, in turn, lead to lost time and wasted resources for health innovators or entrepreneurs as they try to identify problems to solve.

#### Health System Not Receptive to Innovation

A total of 55% (6/11) of innovators or entrepreneurs expressed the theme that the health system is not receptive to innovation. They cited aspects such as not hearing back from representatives within the health system and trying and failing to work with the health system for years and that it is easier to get products into the private areas of the health care system. Not only is the prevailing sentiment that the health system is not receptive to innovation, but comments also showcased innovators or entrepreneurs avoiding the public health system because of this lack of receptivity. One of the innovators or entrepreneurs noted the following:

...we have by design decided to focus our efforts on the private sector, and not the public sector.

P31, innovator or entrepreneur

#### Limited Absorptive Capacity

A total of 50% (7/14) support partners echoed innovators or entrepreneurs’ belief that the health system is not receptive to innovation, commenting that it is not designed to understand its own challenges and adopts technology slowly. They noted that the health system’s role is to provide health care services, not to test new technologies, and suggested some explanations for why the health system is resistant to innovation. Examples of barriers to innovation include the protection of patient privacy and the fact that health systems in Canada are generally focused on multinational companies, encouraging the promotion of a procurement model of technology adoption:

...when you’re starting out and looking to see if something can work, you don’t need to apply a procurement model to it. And unfortunately, that’s what we do in Alberta.

P22, support partner

One of the health system leaders agreed, emphasizing that health systems currently do not have the ability to trial new innovations and do not have the incentives to move away from the current vetted technologies:

We have these products that we feel are our ideal state all the time, and that to detract or maybe push away from innovation or new concepts or new design. Because if we have something that’s vetted and supported then why would we change it even though something might be better?

P24, health system leader

#### Skeptical of New Technology

A total of 29% (2/7) of health system leaders raised this theme, saying that health care workers can be skeptical of new innovations. This is broadly because they can end up being responsible for teaching patients how to use the software, leading to the belief that digital health technology will ultimately make their lives more difficult. Therefore, it is paramount to maintain engagement with clinicians throughout the innovation process to gain their support:

I think the problem that every digital program is up against is it has a bad reputation, right? To the medical staff. It’s a no brainer that it’s better for accuracy and decreasing med errors and that kind of thing. But the reputation it has is this is going to make my life harder, basically. It’s going to...I don’t get paid for it. I’m going to spend more hours getting things done.

P33, health system leader

#### Billing Is a Hidden Problem

Innovators or entrepreneurs expressed the theme that it is difficult to adopt innovation within the health system because of technological barriers, including the use of outdated technology (ie, older operating systems and internet browsers) and the difficulty in integrating novel products into the billing infrastructure:

How do we get it in the doctor’s hands and how do they get compensated for using that tool as part of it? Because we’re operating outside of the norm. Normally, we schedule an appointment, you go in and see your doctor. She spends 30 minutes with us and then codes it to this bill code. So, if I now text her my longitudinal data and she spends 20 minutes assessing that in an application, can she still bill for that? How does that happen?

P18, innovator or entrepreneur

#### Liability of Smallness

In addition, 35% (5/14) of support partners identified the challenges with product integration. These include gaps in knowledge related to the existing solutions between frontline and operations staff in the health system, a lack of bandwidth or capacity to bring innovations into the health system, and the difficulty in getting innovation into health systems without a strong company or clinical reputation. One of the support partners noted the following:

...if you’re a new software startup in Calgary, as an example, [large healthcare organization] probably isn’t going to give you the time of day until you have some sort of reputation.

P06, support partner

Health system leaders also explained that modernizing the health system is a top priority and that there is a mismatch between system operations and what is currently unfolding within the research literature. One of the health system leaders expressed the following:

...sounds so trivial when I say it. The problem that we have been solving and are still solving is just becoming part of the 21st century!

P35, health system leader

#### Create a Solution and They Will Come

In support of the first proposition, 22% (4/18) of funders and support partners, indicated that innovators or entrepreneurs from outside the health care space can try to solve pain points that do not exist or to develop a solution before the problem is clearly defined. One of the support partners described this in the following manner:

...the tendency is to come up with a mouse trap and then look around to see if anyone wants it. The tendency is solution first.

P19, support partner

One of the funders shared their perspective on this:

So I would say for, so with a lot of the companies that we see coming to us from the health sector, a lot of them have novel technology, but aren’t necessarily sure how to commercialize or where the best, business opportunity is for them.

P16, funder

#### Lacking Domain Knowledge

A total of 10% (2/21) of health system leaders and support partners, commented that innovators or entrepreneurs without a health care background can struggle to navigate the health system, especially with regard to procurement:

If it is a truly legitimate problem within the health system, really understanding it, if you’re not within the health system can be challenging. Navigating the procurement.

P32, support partner

There’s no point in a small startup working in a lab, and we do see this all the time and I’m sure you’ve experienced it, they’re in their labs with their techie people and then they bring you this thing and you go, “This is a hospital. No, that wouldn’t work here.” They tend to come from...They’re passionate around technology and not from that hospital or healthcare operational understanding. I think if more of those companies had that sort of engagement and advice early on, they’d be creating better products too.

P35, health system leader

#### Use Key Informants

However, 45% (5/11) of innovators or entrepreneurs stated that they seek the help of a clinical adviser or have personal access to a clinician to help identify problems or evaluate the usefulness of innovations.

#### Health System Could Publish Problems

A total of 18% (2/11) of innovators or entrepreneurs and 14% (1/7) of health system leaders suggested that health systems could aid innovation by publishing their needs using a portal or website. One of the innovators or entrepreneurs mentioned that this method had already worked successfully for them in another country:

In a hospital in Berlin, because we did some testing with them, and they effectively published...I don’t know if it’s a hospital, or maybe it’s some medical association, but they published their most critical use cases. Or maybe areas where they need technology to help. I know there is in Berlin. Anybody has access to it, and they get an idea...And they even have a contact, that if you want to understand a little bit more you can talk to somebody.

P02, innovator or entrepreneur

#### Ways Health System Could Identify Problems

Moreover, 9% (1/11) of innovators or entrepreneurs and 7% (1/14) of support partners suggested 2 ways in which the health system could better identify problems: surveying clinicians to identify their needs and documenting clinical workflows so that innovators or entrepreneurs can see the current process and imagine an improvement:

If the technical as well as the clinical leaders had a list of things that they’re wasting time on or things that they would love to embrace. If some of that was documented, I feel that would be golden for some of us in the industry in order to really feed off of them.

P11, innovator or entrepreneur

### Proposition 2: Digital Health Innovators and Entrepreneurs Struggle to Access Relevant Data

We anticipated that digital health innovators or entrepreneurs would generally be unaware of where to get access to the data they need to validate and test their innovations and would be uncertain how to access and manage the data when they did locate them.

#### Clinical Data Access Is Closed

Supporting the second proposition, 45% (5/11) of innovators or entrepreneurs mentioned that they faced challenges accessing the data needed to support their innovation journey. One of the innovators or entrepreneurs stated the following:

In healthcare, you find lots of challenges. The first challenge is you don’t get any access anywhere. No data access, you have nothing.

P03, innovator or entrepreneur

#### Easier to Access Data in Other Jurisdictions

Support partners and health system leaders echoed the data needs of innovators or entrepreneurs. They commented that, in general, health systems could improve access to their data for innovators or entrepreneurs, as has been done in other regions.

Other jurisdictions have anonymized data in a way that is sufficient enough to provide access to patient data. Why can’t we do it? Why can’t we just follow one of the models that have already been established as successful in other jurisdictions?

P32, support partner

#### No Visibility to Process or Administrative Data

Most commonly, innovators or entrepreneurs said that they would benefit from more data to assess the market for their products. They mentioned that these data may be used to assess details such as the prevalence of clinic visits and the necessary information that would be valuable to clinicians when making decisions. A total of 11% (4/36) of participants noted that these additional data would allow them to better understand the potential growth of their innovations. One of the innovators or entrepreneurs said the following:

I think having access to administrative hospital data, so you can link your very specific data collection with general hospitalization data, because I think it would speak to the ability to expand.

P15, innovator or entrepreneur

#### What Is in Range and Out of Range

In addition, innovators or entrepreneurs commented that they would benefit from knowing what metrics the health system requires. For example, an innovator or entrepreneur highlighted the difficulty in knowing what the normal versus critical biometrics are as well as how accurate measurements should be:

...we have to find out to understand if the condition is critical or not? How accurate it should be, specifically during those critical events?

P02, innovator or entrepreneur

Although innovators or entrepreneurs typically had more access to data than anticipated, they still expressed a need for additional data access to better market and scale their products. Furthermore, the consensus among support partners and health system leaders was that the innovation ecosystem would be enhanced by allowing innovators or entrepreneurs more access to health system data, not simply clinical data but also administrative and process data.

### Proposition 3: Digital Health Innovators and Entrepreneurs Struggle to Understand Where to Start and Where They Need to Go on Their Innovation Journey

We anticipated that digital health innovators or entrepreneurs would face challenges in knowing where to start and how to get help with their innovations and that this challenge would be more pronounced for innovators or entrepreneurs without experience in the health system.

#### Not Sure Where to Start

Overall, we found strong support for our proposition that innovators or entrepreneurs struggle to navigate the digital health ecosystem. A total of 47% (17/36) of participants (innovators or entrepreneurs, support partners, health system leaders, and funders) referenced the theme that innovators or entrepreneurs require more guidance through the digital health ecosystem. From the innovator or entrepreneur perspective, 55% (6/11) of participants expressed that they were unsure where they could get assistance and that they would have benefited from help earlier on in their innovation journey:

We could have saved ourselves a lot of time, money, and effort if someone had said, ‘Okay, yeah, you guys are here, but maybe you should be doing this,’ And not someone who is advising us for their own gains.

P08, innovator or entrepreneur

#### “Outsiders” (Nonhealth) Are Excluded From the Ecosystem

Echoing the comments from innovators or entrepreneurs, 50% (7/14) of support partners presented the idea that the ecosystem is difficult for those starting from outside the health care system to understand, lacks a clear path forward for innovators or entrepreneurs, and has overlaps between support providers that force innovators or entrepreneurs to decide between multiple options:

They receive calls for expressions of interest from 10 different organizations and then kinda go okay well I can’t work with all. I don’t have the capacity cause I’m a start up. This one sounds interesting but I don’t know how it connects to the rest of them and I don’t know that I have an overall navigator saying we should go through this first and this and this. Then they get the result from whatever platform they work with, and they don’t know what the next step might be.

P01, support partner

#### The Path to Innovation Is Obscured

These issues are apparent to health system leaders, with 43% (3/7) of them saying that innovators or entrepreneurs need better guidance and that the process of getting innovation into the health system could be more transparent:

I think having a clear process, which we’re trying to move to, and like [my colleague] said, it’s taken us 20 years and it’s not perfect. We’re trying to get there. It helps the innovators too, because I think it should help the innovators and those companies and things because they can see the process. They can also see if a decision maybe hasn’t gone the way that they wanted, who did it? How to appeal or discuss.

P25, health system leader

#### Support Is Uncoordinated

In addition to the navigation challenges that innovators or entrepreneurs face, the digital health support ecosystem currently relies on ad hoc interactions between support partners and could be better connected. A total of 44% (11/25) of participants—a mix of funders, health system leaders, and support partners—indicated that the cooperation between ecosystem support partners was informal. Their comments centered on the perception that the support ecosystem requires more collaboration. This is because of the reliance on unstructured referrals between support partners, who make recommendations based on personal knowledge that can be lost when these individuals leave the support organization:

I hold a lot of relationships already and let alone two years from now. And it’s kind of the story of these types of roles is that, those relationships basically have to start again from scratch because someone leaves their role.

P10, support partner

Although the support partner ecosystem remains disconnected, the ecosystem members are aware of this problem and want to improve collaboration. Overall, 33% (6/18) of participants, including both funders and support partners, made the point that the ecosystem needs more collaboration, with 3 support partners suggesting not only that there needs to be better handoffs between support partners but also that they are, in fact, eager to collaborate more:

I think that my personal view is that we have major holes in this game here. I think that we have many, many different organizations that need to work a lot better together and have you know better handoffs and better synergy between the service offerings.

P01, support partner

#### Navigating Funding Is Challenging

One of the funders pointed out that it is not clear which organization provides funding at which stage. Another funder said that venture capitalists do not have the technical ability to evaluate start-ups that are subject to regulations. However, investors are attracted to the shorter timelines required to commercialize digital health products. One of the support partners noted that innovators need to be aware of the due diligence requirements that funders will require and that innovators are often unaware of these and do not adequately think about them in advance.

#### Ecosystem Needs More Funding at All Stages

A total of 27% (3/11) of innovators or entrepreneurs, 14% (2/14) of support partners, and 25% (1/4) of funders raised the theme that early-stage funding is lacking in the digital health ecosystem. Innovators or entrepreneurs mentioned that additional early-stage funding could be used to help them develop business plans and improve growth. Support partners mentioned that early-stage funding is lacking overall and that innovators or entrepreneurs are often unsure of what the exact requirements are for funding during the early stages. One of the funders emphasized the notable gap that Alberta does not have any incubators or accelerators with attached funding:

...we don’t have any incubators or accelerators that have funding attached to them, which is typical in Ontario, Quebec and British Columbia.

P20, funder

Moreover, 8% (2/25) of innovators or entrepreneurs and support partners described the theme that the ecosystem is lacking funding at all stages. From the innovators or entrepreneurs’ perspective, raising funds is difficult, often requiring help from friends or family, and there need to be better connections with angel investors or venture capital firms. This support partner mentioned that the most common request they receive is to assist in finding funders or grants:

...raising money is very, very difficult. You have to have an investor community that’s willing to take a chance. And for us, it was friends, families, some local angels.

P21, innovator or entrepreneur

#### Easier to Navigate Ecosystem in Other Countries

In addition, 50% (2/4) of support partners and 9% (1/11) of innovators or entrepreneurs expressed that it is difficult to get products sold in Canada, relative to other countries, owing to regulations and a lack of capital:

...it’s like a cultural risk aversion for companies to try out new solutions or solutions from younger, smaller companies. So they tend to get their first customers outside of Alberta and outside of Canada.

P19, support partner

Overall, navigating the digital health ecosystem in Alberta is challenging for innovators or entrepreneurs. However, this need is recognized by ecosystem players who have the desire for better collaboration between support partners, including more transparency around the available funding.

### Proposition 4: The Breadth of Training Provided Does Not Adequately Meet the Needs of Digital Health Innovators and Entrepreneurs

We anticipated that digital health innovators or entrepreneurs would need a variety of training specifically tailored to the unique needs of the digital health industry.

#### Business Training

A total of 53% (19/36) of participants referenced areas where innovators or entrepreneurs could use additional training in the digital health ecosystem. The most prevalent area of need was business training, with 10 references from innovators or entrepreneurs, support partners, and funders to areas of need such as general business training, marketing, and pitching ideas to investors. Business training was also an area of need for clinical innovators or entrepreneurs, with 36% (4/11) of participants referencing their need for training to better understand app development or get their product to market:

We both come from the researcher side, so we have that squared away, but just trying to integrate that, and our research ideas into the actual business and money side of it, getting the product out there into the market.

P15, innovator or entrepreneur

The other piece that we don’t have much training in and bluntly, it is something that I don’t really blame the clinical group for not knowing, but they don’t understand what the operation side is. For all they have to submit, paperwork or somebody signs off on their budget, they don’t really understand the black box know of the inner workings. And as somebody who’s been learning that on the go over the past little while, it is not complicated, but if you’ve never had it opened up, you don’t understand the rules and things that supply chain management works under.

P25, health system leader

#### Privacy Training

Although business training was a prominent focus of innovator or entrepreneur recommendations, regulatory or privacy training was identified as a need by 14% (2/14) of support partners and 25% (1/4) of funders, and training in health economics was identified as a need by 14% (1/7) of health system leaders and 7% (1/14) of support partners. Innovators or entrepreneurs’ training needs are ubiquitous and diverse, highlighting the need for a customized referral process and improved collaboration between the existing training providers. A total of 29% (2/7) of health system leaders and 14% (2/14) of support partners mentioned that privacy regulations are a barrier to innovation because they restrict access to data and that privacy regulations can be surprising to innovators or entrepreneurs who are unfamiliar with the requirements:

I think that people are becoming more savvy to the issue of privacy and healthcare information sensitivity. But that is still something that surprises some people in terms of what kinds of safeguards they need to have in place, and why they can’t just be the direct link between a doctor’s office, who may have results, and the individual.

P06, support partner

#### Regulatory Training

However, 29% (2/7) of health system leaders mentioned that while privacy regulations are a challenge to innovation, the public wants more access to their data and is less concerned about privacy. Innovation could be improved by allowing more access to patient data, which is something that the general public is becoming more comfortable with:

Every time we do these surveys across Canada, the public expects their data is going to be available across their healthcare providers and within their circle of care, yet we haven’t facilitated that in any way. We’ve actually made it almost impossible. I think we need more opportunities for interoperability data exchange, whether it’s through data sharing agreements or some reworking of health information custodians.

P35, health system leader

### Proposition 5: Digital Health Innovators and Entrepreneurs Lack a “Space” to Evaluate Their Offerings

We hypothesized that digital health innovators or entrepreneurs lacked locations to evaluate and validate their digital health products. A total of 82% (9/11) of innovators or entrepreneurs and 14% (2/14) of support partners, identified themes related to the validation needs of innovators or entrepreneurs.

#### Physical Living Laboratory Location

Overall, 36% (4/11) of innovators or entrepreneurs mentioned the need for a designated place to validate that a product works and is safe to use. This validation involves testing the accuracy, reliability, and safety of a device:

You need that place where you take something, you build a functional unit, but then you need to really vet it. Does it really work? Does it really do what you intend it to do? And did you understand the requirements correctly.

P15, innovator or entrepreneur

#### Simulated Location

A distinction was also made between a physical space where innovations and products could be evaluated in a real-world “living laboratory” setting and a simulated environment. Several support partners noted the following:

I know with [large health organization], they’re also working on this big synthetic data initiative. I don’t know if you’ve heard of that, but that’s certainly an opportunity. It’s a new thing, but it seems quite promising and it seems like it actually generates the kind of data that a machine learning algorithm would actually be able to be trained on. That being said, if your machine learning algorithm requires actual images, diagnostic images, it’s a little bit different than just lab results. But nevertheless, it’s hard to create synthetic data around those kinds of complicated bits of data. But certainly you can start somewhere, get the ball rolling, and then as people become more comfortable with it, start prying open those doors.

P32, support partner

I think simulations have a big role to play here and is something that you could easily build in a very confidential manner of, here’s a simulated, it’s not a real. And whether you do that through low tech or high tech, you could do it with actors in a room. ‘This is how I would talk to a patient. This is why I couldn’t ask them. This is that.’ But you could also do it as virtual simulations, where you’re basically recreating a simulated environment. ‘This is what it’s like at the average maternity ward in Alberta.’ Or maternity ward’s over 20 beds say. ‘When we talk about this being a problem, here’s a picture of the thing that is a problem.’ And you can immediately say why.

P34, support partner

#### Advice Regarding Options

A total of 27% (3/11) of innovators or entrepreneurs and 7% (1/14) of support partners identified the need for advice regarding validation possibilities and potential next steps. This need involves identifying the type of validation an innovator or entrepreneur might need. For example, the level of rigor of a clinical trial is compared with that of the validation of a minimum viable product or initial prototype used to economically validate key business and clinical assumptions:

Knowing the different options, because obviously a more rigorous clinical trial would be more expensive. Sometimes mobile-held applicants don’t require that compared to a medical device. So being able to know what the minimum threshold might be, would be useful in terms of being efficient with funding.

P14, innovator or entrepreneur

#### Local Validation for Local Context

In addition, 27% (3/11) of innovators or entrepreneurs and 7% (1/14) of support partners discussed the theme of needing testing, either in general or locally, representing innovators or entrepreneurs’ need for better access to local validation to help them demonstrate value of their interventions to the health system:

International validation isn’t enough for them, right? They want to be able to see local proof of concept. They want to be able to see people who they know, who are familiar to them put a seal of faith on these technologies.

P31, innovator or entrepreneur

#### Validation for Scaling

A total of 36% (4/11) of innovators or entrepreneurs expressed the need for more validation with clinicians or in clinics. Innovators or entrepreneurs spoke about the benefits of observing clinical workflows, validating the use case with clinicians, and having access to clinicians who can give advice to improve the product:

Definitely, can we call them subject matter experts? So, it’s people who really understand field. Because you can read a lot of papers, but if you still don’t know what it all means, and it’s hard to learn everything, you will still end up building a product that doesn’t have good applicability.

P02, innovator or entrepreneur

Furthermore, 18% (2/11) of innovators or entrepreneurs communicated the need to validate their product with the end users to verify usability or adherence. Innovators or entrepreneurs need to ensure that their products are something that a clinician or a member of the general public would feel comfortable using:

...the challenge I’m going to have on the consumer side is consumer perception about what we’re doing. Are they going to use the product?

P18, innovator or entrepreneur

#### Validation Pathway Into the Health System

Overall, 14% (2/14) of support partners mentioned that it is difficult for innovators or entrepreneurs to validate their products within the health system because of its size and the lack of a formalized mechanism to do so:

...a more formalized mechanism to participate in those validation opportunities with healthcare providers and healthcare organizations [is needed].

P05, support partner

## Discussion

### Principal Findings

Our exploration of the systemic conditions within a digital health ecosystem for key stakeholders, including digital health innovators or entrepreneurs, investors, and health system leaders, illustrates the role of these conditions in predominantly determining the success of the ecosystem. Significant gaps were identified in each component of the systemic conditions within the digital health ecosystem of Alberta. However, the most foundational gap appears to be in the context of navigating the ecosystem in the absence of intermediaries tasked with providing guidance to digital health entrepreneurs around the available support services and dependencies among the various ecosystem actors and programs offered. These findings provide support for the underlying premise of the HIPP program, which motivated this study, to incentivize greater coordination among ecosystem support providers.

### Comparison With Prior Work

Our findings add to the emerging literature on ecosystems [3], specifically within a digital health context [6]. The set of propositions guiding this study was developed from the literature; however, our findings provide additional, often nuanced, insights into each of these themes. For accessing real problems, we support a prior work that identifies this issue, specifically within the Alberta context [7]. The Alberta SCNs created in 2012 have a mandate for consolidating the real problems they are facing and ranking them for use by the ecosystem. However, as the findings from this study show, a decade into that mandate, there remain concerns over the transparency of those problems, as they do not appear to be well known or understood within the digital health entrepreneurial community. The SCNs are by their very nature not consumer focused, even if they continue to deliver on increasing the accessibility and transparency of the real problems they face, leaving a significant gap on the consumer and prevention side of the identification of real problems. In many respects, our findings parallel, at least at the leadership and operational levels, the challenges identified in Ontario [6].

Within the context of information, we provide further evidence of the challenges in accessing clinical and administrative data [12]. In Alberta, great strides have been made with respect to administrative data access, and initiatives at both regional and provincial levels have streamlined this access. However, transparency of these processes remains a challenge for many innovators or entrepreneurs. From the perception of our participants, it is often more dependent on the informal process of finding a contact that takes an interest in your project to help you navigate the administrative burdens of discovering what data are available and how to request access to them.

This notion of challenges around navigation was a recurring theme across every proposition. This issue was particularly acute for funding opportunities within the digital health ecosystem of Alberta. An issue that was highlighted in this study is the unique challenges that innovators or entrepreneurs from outside health care face when transitioning into the health care industry. This is particularly disconcerting in Alberta, where health care is presented as a destination industry for individuals in sectors that are in decline to repurpose their talents and experience. This connects to the broader discussion of certain groups being excluded from a digital health ecosystem because of a lack of necessary infrastructure, social disadvantage, economic factors, health status, lack of skills or interest, or inadequate recognition of their needs [11].

Although policy makers have recognized the lack of coordination within ecosystems and have attempted to provide concierge services, one-stop points of contact, and centralized triage for innovators or entrepreneurs, these have not generally been successful. However, the findings from this study and our experience with the HIPP program provide additional insights into the design criteria for such navigation services in the future. Specifically, it is nearly impossible to centralize such services because no organization or government holds a sufficient mandate or resources to do so. For example, a program spearheaded at the federal level could easily be undermined by a lack of provincial or municipal referrals. The HIPP approach provided a key insight in that the incentives were structured such that the participants were rewarded for not only delivering the services they promised but also contributing to benefits that accrued to the ecosystem overall. Thus, balancing group and individual incentives is essential for successful coordination, as the group or ecosystem-level incentives are generally lacking. These incentives need to be flexible to allow for the broad inclusion of stakeholders, and they also need to incentivize a reduction in duplication. Currently, all incentives are for support providers to deliver as many services as possible without regard for how well they can deliver those services, whether those services integrate with the services of other providers, or whether such services are already better deployed by other ecosystem stakeholders. For organic ecosystem coordination to flourish, there needs to be equal incentives to give up or better integrate services as there is to create new services. Finally, a common approach to ecosystem orchestration is the use of web-based platforms that attempt to document all the services offered by the ecosystem combined with navigation tools to help innovators or entrepreneurs self-service at least a portion of their search and selection processes. There are prominent examples of these at the provincial (eg, Alberta Innovates’ Support Finder) and federal (eg, Government of Canada’s Business Benefits Finder) levels, where such data aggregation is attempted, but they usually suffer the same fate after the initial funding runs out—the data quickly become stale, and the usefulness rapidly declines, or they only cover parts of the ecosystem that are relevant to the sponsor’s mandate. A successful approach would require a governance structure in which such platforms are not owned by a specific ecosystem stakeholder; instead, the platform should be owned by a consortium of ecosystem players. This greatly aligns the incentives and ability to coordinate activities in a structured manner and keep data current. Such 2-sided markets are still challenging to maintain, but incentivizing the supply side (ie, support providers) to cooperate quickly leads to reasons for the demand side (ie, innovators or entrepreneurs) to show up, which, in turn, reinforces the supply side.

On the training side, one approach to address this gap is to provide differentiated training options for medical insiders versus outsiders. Medical insiders generally needed business and privacy or regulatory training, while medical outsiders needed additional training on the medical system, billing, and procurement. Interestingly, being an insider did not always accompany an understanding of some of these topics, so some level of baselining is needed to assess digital health innovators or entrepreneurs’ readiness.

Finally, there is a need for evidence supporting the claims that digital health offerings provide real improvements over the status quo [16]. Of particular interest to W21C, as a support provider located within an AMC, are the future roles of AMCs in supporting digital health ecosystems. AMCs provide cross-disciplinary expertise in health and technology and train the next generation of health professionals, thus providing the opportunity to connect academics and clinicians across a variety of disciplines with innovators and entrepreneurs [15]. Demand for both living laboratories and simulated environments is strong among digital health innovators or entrepreneurs. The ability to evaluate their processes, algorithms, devices, and software in an environment that provides systematic feedback is essential [14]. This issue was particularly acute for digital health innovators or entrepreneurs, as one of the advantages of digital health innovations is the rapid rate at which they can be scaled. This is in sharp contrast to many health care innovations such as drugs, biomedical interventions, and medical devices, which are subject to substantially more regulatory requirements [12].

### Limitations

The findings of this study are based on the experience of the digital health ecosystem in Alberta, Canada, which may not be generalizable to other contexts. However, as discussed in the comparison with prior work, there are common themes that appear to transcend jurisdictions. Although the research team selected ecosystem actors who broadly represent several factors of theoretical importance, there is a possibility that key stakeholder perspectives were omitted.

### Conclusions

Navigating the systemic conditions of the digital health ecosystem is extremely challenging for innovators or entrepreneurs without prior health care experience, and this remains an issue even for those with such experience. Policy interventions aimed at increasing collaboration among ecosystem support providers, along with tools and incentives to ensure coordination, are essential as ecosystems grow. By improving the systemic conditions highlighted in this study, the Alberta digital health ecosystem can increase its competitiveness and foster greater innovation, talent, opportunities, choices, and access to digital health care across the country [21].

## Abbreviations

AMC: Academic Medical Center
HIPP: Health Innovation Platform Partnerships
SCN: Strategic Clinical Network
W21C: Ward of the 21st Century

## Data Availability

The data sets generated and analyzed during this study are available from the corresponding author upon reasonable request.
